# The effect of prognostic nutritional index on diabetic patients with myocardial infarction

**DOI:** 10.1186/s13098-024-01409-6

**Published:** 2024-07-27

**Authors:** Yanchun Peng, Aijie Lin, Baolin Luo, Liangwan Chen, Yanjuan Lin

**Affiliations:** 1 Department of Nursing, Fujian Medical University Union Hospital, No. 29 Xinquan Road, Fuzhou, 350001 Fujian Province China; 2 School of Nursing, Fujian Medical University, No. 1 Xuefu North Road, University Town, Fuzhou, 350122 Fujian Province China; 3 Department of Cardiac Surgery, Fujian Medical University Union Hospital, No. 29 Xinquan Road, Fuzhou, 350001 Fujian Province China; 4 Department of Cardiac Surgery Nursing, Fujian Medical University Union Hospital, Fuzhou, China

**Keywords:** Prognostic nutritional index, Diabetes mellitus, Myocardial infarction, Prognosis

## Abstract

**Background:**

The prognostic nutritional index (PNI), a simple and comprehensive predictor of nutritional and immunological health, is connected to cancer and cardiovascular disease. The effects of PNI on myocardial infarction (MI) in individuals with diabetes remain unclear. Thus, we aim to investigate the correlation of PNI with predictive outcomes in this specific population group to inform therapeutic decision-making.

**Methods:**

This prospective observational study included 417 diabetic patients with MI who underwent coronary angiography intervention at Fujian Medical University Union Hospital from May 2017 to May 2020. We collected follow-up and prognostic data from these patients at 6, 12, 18, and 24 months post-procedure via outpatient visits or phone interviews. The main focus of the study was on major adverse cardiovascular events (MACE) in the two years after surgery. Based on the median PNI, patients were categorized into two groups: high PNI (H-PNI) and low PNI (L-PNI). Data were analyzed using IBM SPSS 25.0. Kalpan-Meier survival curves and Cox proportional hazards regression analysis were utilized to examine the associations between preoperative PNI and the prognosis of diabetic patients with MI.

**Results:**

In the study, 417 participants were observed for two years. Of these patients, 159 (38.1%) had MACE. According to the Kaplan–Meier curves, patients in the L-PNI group had more MACE than those in the H-PNI group (log-rank *p* < 0.001) and had a heightened susceptibility to all categories of MACE. After adjusting for confounding variables, the corrected hazard ratio for developing unstable angina in the L-PNI group was 2.55 (95% CI 1.57–4.14, *p* < 0.001).

**Conclusion:**

Low PNI levels are associated with MACE after coronary angiography intervention in diabetic patients with myocardial infarction. This highlights the prognostic value of PNI and broadens its potential use in larger populations.

*Trial registration*: Not applicable.

**Supplementary Information:**

The online version contains supplementary material available at 10.1186/s13098-024-01409-6.

## Introduction

Globally, the incidence of cardiovascular disease (CVD) is reported to have risen from 271 million cases in 1990 to 523 million cases in 2019 [1]. In China, the number of CVD patients reaches 330 million, and CVD accounts for the first place in the composition of urban and rural deaths. The burden it brings to the society is still increasing [2]. Coronary heart disease (CHD) ranks second in prevalence among all CVDs, which includes acute coronary syndromes (ACS). Over 7 million people worldwide are diagnosed with ACS annually, with myocardial infarction (MI) making up over 30% of these cases [3]. Diabetes mellitus (DM) is one of the independent risk factors for CVD. A German study revealed the proportion of acute MI patients with DM grew from 29.8% in 2005 to 30.7% in 2016, showing a general upward trend [4]. In these patients with both conditions, diabetes mellitus worsens systemic inflammation, leading to accelerated catabolism and decreased albumin levels. Patients are more prone to serious cardiovascular events due to inflammation-promoting atherogenesis [5, 6]. As data in most reported current studies, the mortality of diabetic patients with acute MI is one to two times higher than that of non-diabetic patients [7, 8]. Therefore, investigating the relevant prognostic indicators in diabetic patients with MI can predict the progression of the disease and help to reduce the mortality in this group of patients.

Malnutrition lowers immune function and increases inflammation, resulting in complications and worsening illness outcomes. Prognostic nutritional index (PNI) is a novel nutritional index in recent years, first applied by Buzby et al. [9] in gastrointestinal surgery, consisting of serum albumin and total lymphocyte count. Compared with the geriatric nutritional risk index (GNRI), controlling nutritional status (CONUT) score, and mini-nutritional assessment (MNA), the PNI not only assesses the nutritional status of the organism but also effectively reflects inflammatory and immune status. Its predictive capacity has been firmly demonstrated in malignant tumors [10], heart failure [11], and DM [12]. In addition, serum albumin and lymphocytes, which determine PNI levels, are also closely related to MI [13–15]. What's more, some studies have confirmed that about half of the relationship between lymphocyte-related markers and cardiovascular events may be mediated through factors such as DM [16]. Available findings point out a correlation between a reduction in PNI and the increased risk of long-term mortality in acute coronary artery syndrome patients undergoing coronary intervention [17]. Chen et al. [18] discovered that combining the PNI and the Global Registry of Acute Coronary Events (GRACE) score led to a more accurate prediction of long-term all-cause death compared to using the GRACE score alone. The results of a study in patients with CHD showed that DM complicated with CHD exacerbated the poor prognostic outcome of patients with low PNI [19]. Despite mounting evidence documenting the relation of PNI and DM complicated with CHD, the extent of risk regarding PNI in predicting prognosis in diabetic patients with MI is unclear. Further research is necessary to clarify their correlation and ascertain the potentially more favorable predictive capability of PNI.

Therefore, this study focused on diabetic patients with MI to investigate the role of nutritional and immune status, as assessed by the PNI, in the prognosis of this population and to provide guidance and a basis for clinical decision-making.

## Methods

### Study design setting and population

In this study, a prospective observational study method was used to select 417 diabetic patients with MI who underwent coronary angiography intervention at Fujian Medical University Union Hospital between May 2017 and May 2020. The hospital’s cardiac surgeons diagnosed MI in all patients based on established guidelines, clinical symptoms, blood tests, and imaging data. The diagnosis was documented in the patient's electronic medical record system and later accessed by the researchers. The patients with myocardial infarction were diagnosed according to the diagnostic criteria outlined in the third and fourth universal definitions of MI [20, 21]. Patients of this study represented a population of men and women aged ≥ 18 years who gave informed consent for inclusion. Exclusion criteria were end-stage liver or kidney disease, systemic inflammatory response syndrome, history of cancer, missing preoperative serum albumin, and absolute lymphocyte count data. We divided the patients included in the study into two groups based on the median preoperative PNI of the entire study population. Patients with PNI levels above this value were allocated to the high PNI (H-PNI) group, while those below this value were allocated to the low PNI (L-PNI) group. The study protocol adhered to the ethical principles outlined in the 1975 Declaration of Helsinki, and prior authorization for conducting research with human subjects was acquired from the Ethics Committee of Fujian Medical University Union Hospital (No. 2020KY0127). Each patient participating in the study provided written informed permission.

### Data collection

Data were extracted from the electronic clinical records. The general baseline information mainly included demographic characteristics (e.g., sex, age, body mass index [BMI]), medical history and clinical condition (e.g., hypertension, hyperlipidemia, previous thrombosis, pulmonary hypertension, pulmonary infections, smoking, drinking, previous percutaneous coronary intervention [PCI], previous percutaneous transluminal coronary angioplasty [PTCA], previous cardiac surgeries, previous cardiac bypass), medication, laboratory examination (e.g., serum albumin [ALB], absolute lymphocyte count [ALC], C-reactive protein [CRP], fasting blood glucose [FBG], glycosylated hemoglobin, type A1c [HbA1c], creatinine [Cr], uric acid [UA], B-type brain natriuretic peptide [BNP], serum triglycerides [TG], left ventricular ejection fraction [LVEF]), angiographic data (e.g., repeat imaging interval, infarcted vessel type and number). The procedure for collecting blood samples for biochemical tests in this study was as follows: the nurse in charge told the patients to fast and abstain from food and drink after 8:00 p.m. on the night of admission, and then collect blood samples from 6:00 a.m. to 8:00 a.m. on the next day, and fast and abstain from food and drink for at least 10 h for each patient.

### Outcomes and follow-up

The effect of preoperative PNI on the prognosis of diabetic patients with MI two years after the end of coronary angiography intervention was analyzed. Patients were followed up on the first day after intervention. Follow-up data were monitored and recorded by nurse practitioners and master's degree nursing students through outpatient interviews and telephone at 6, 12, 18, and 24 months. The primary outcome of this study was the composite outcome of the first occurrence of major adverse cardiovascular events (MACE) after coronary angiography intervention. The outcome events included cardiovascular death, nonfatal stroke, arrhythmia, admission for unstable angina, or heart failure. Also, the diagnosis of atrial fibrillation, heart failure, stroke, and unstable angina for the outcome event was standardized using the disease definitions in code ICD-9. After the patient's first MACE (except for death due to CVD), follow-up was continued to record whether the patient had a recurrence of MACE until the end of the 2-year follow-up period.

### Study definitions

The calculation of BMI is weight/height^2^. BMI < 18.5 was considered underweight; BMI ≥ 18.5 kg/m^2^ and BMI < 24.0 kg/m^2^ were considered normal; BMI ≥ 24.0 kg/m^2^ and BMI < 28.0 kg/m^2^ were considered overweight; BMI ≥ 28.0 kg/m^2^ was defined as obesity. DM was defined as asymptomatic individuals with more than two FBG ≥ 7.0 mmol/L, oral glucose tolerance test (OGTT) 2 h postprandial blood glucose ≥ 11.1 mmol/L, HbA1c ≥ 6.5%, typical hyperglycemic symptoms or hyperglycemic crisis and random blood glucose of 11.1 mmol/L, previous diagnosis of DM, or oral antidiabetic medication or insulin treatment [22]. High blood pressure was diagnosed if one of the following criteria was met: repeated blood pressure measurements in the hospital with systolic blood pressure ≥ 140 mmHg, diastolic blood pressure ≥ 90 mmHg, antihypertensive medication use, and self-reported hypertension [23]. Dyslipidemia was defined as total cholesterol ≥ 6.20 mmol/L, TG ≥ 2.25 mmol/L, low density lipoprotein cholesterol ≥ 4.13 mmol/L, or high density lipoprotein cholesterol < 1.03 mmol/L [24]. The 1997 World Health Organization definition of smoking was used: people who had smoked continuously for a cumulative period of 6 months or more in their lifetime. According to a related study, smoking cessation for more than 15 years can repair endothelial damage in coronary arteries [25]. In this study, individuals who had refrained from smoking for over 15 years were classified as nonsmokers, whereas those who had abstained for less than 15 years were classified as smokers. The definition of alcohol consumption is average alcohol consumption ≥ 100 g per week for more than one year before admission [26]. PNI was calculated as 10 × ALB (g/L) + 5 × ALC (× 10^9^/L).

### Statistical analysis

Patients were categorized into two groups by median PNI. Continuously normally distributed data were summarized as mean ± standard deviation using t-tests; non-normally distributed continuous data were summarized as median with interquartile range using the Mann–Whitney U test. Categorical data were expressed as proportions, and the χ^2^ test was used. Survival curves were plotted using the Kaplan–Meier method, and the incidence of MACE in both groups was tested using the log-rank test. In Cox proportional risk regression analysis, the association of preoperative PNI status and clinical outcomes was expressed by estimating hazard ratios (HRs) and 95% confidence intervals (CIs). Potential confounders were analyzed by adjusting for age, LVEF, hypertension, hyperlipidemia, CRP, Cr, pulmonary infection, and smoking based on the results of univariate analysis. Statistical analyses were performed using SPSS statistical software version 25.0 (IBM Corp, Armonk, NY, USA). Figures were created using GraphPad Prism version 8.3 (GraphPad Software, San Diego, California, USA). The *p*-value of less than 0.05 was considered statistically significant for all analyses.

## Results

### Baseline characteristics

**Fig. 1:**
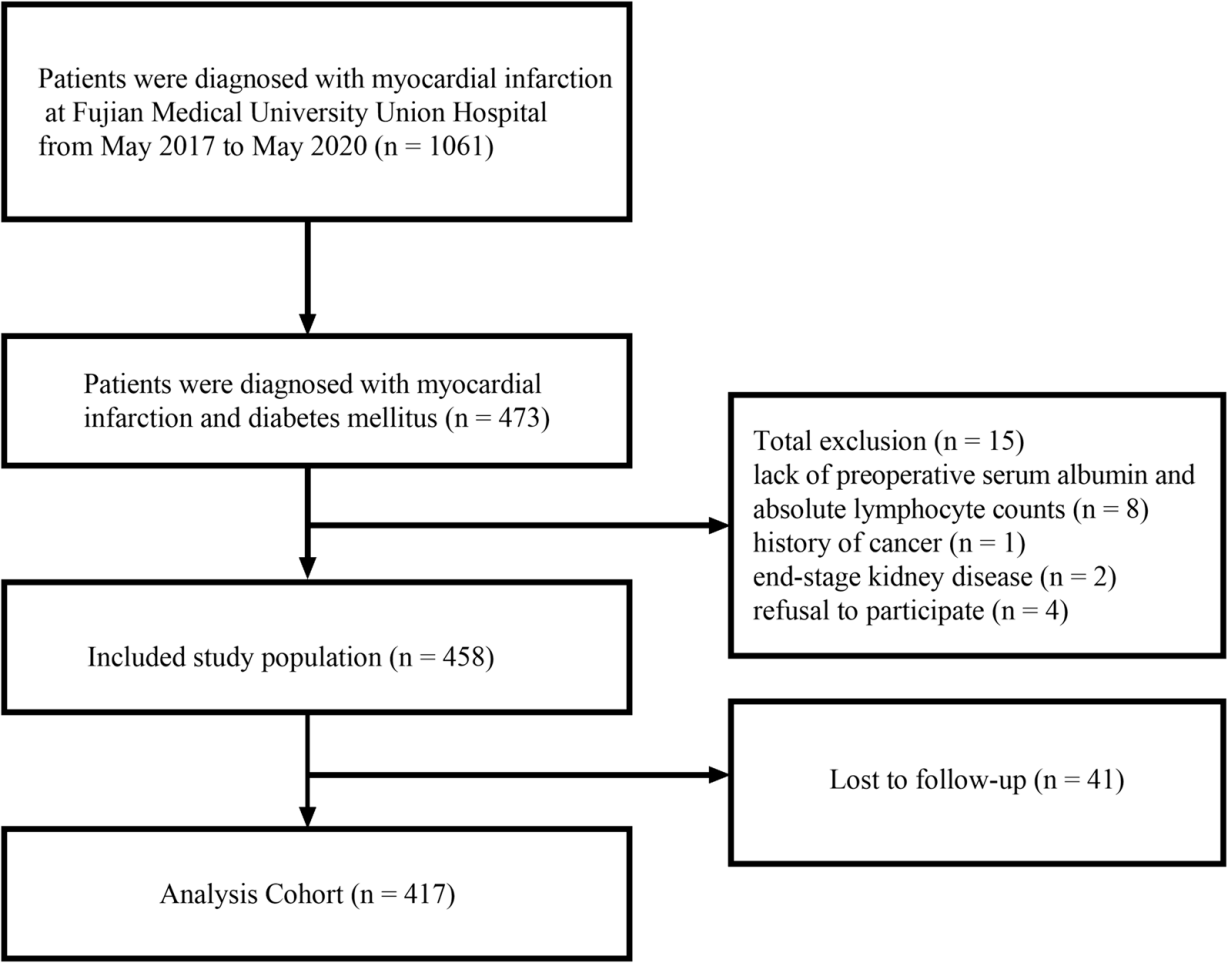
Flow diagram of patients in the study

Data on 458 diabetic patients with MI who met the inclusion criteria were included in this study, as indicated in Fig. 1. Fifteen participants were filtered out based on the inclusive and exclusive criteria, and another 41 patients were lost during the 2-year follow-up, resulting in 417 patients (91.05%) being included in the final analysis. During the follow-up period, there were 15 cardiovascular deaths, 40 cases of atrial fibrillation, 20 cases of heart failure, 40 cases of non-fatal stroke, and 88 cases of unstable angina.

The baseline demographics were sorted by MACE occurrence. The study population had a mean age of 64.87 ± 10.46 years, with 328 males (78.7%). Patients with MACE exhibited significant differences in ALC, ALB, preoperative PNI, age, BMI, CRP, TG, presence of comorbidities (hypertension, hyperlipidemia, pulmonary infections), history of prior PCI, smoking status, LVEF, Cr, and intervention programs, in comparison to those without MACE. (Additional file 1: Table S1).

The baseline characteristics of the two patient groups are presented in Table 1, categorized by preoperative PNI level (median 47.3). Patients on L-PNI comprised 49.9% of the total sample. Age, BMI, comorbidities (hypertension, hyperlipidemia, pulmonary infection), LVEF, Cr, CRP, TG, and time between imaging examinations were all significantly different between the two groups.

**Table 1:** Baseline characteristics categorized by preoperative PNI levels

Variable	H-PNI (n = 209)	L-PNI (n = 208)	*p*
Demographic characteristics			
Sex, male	173 (82.8)	155 (74.5)	0.400
Age, years	62.4 ± 10.3	67.4 ± 10.0	< 0.001
BMI, kg/m^2^	24.5 (22.9, 26.2)	23.9 (21.6, 25.5)	0.004
Medical history and clinical condition			
Hypertension	132 (63.2)	152 (73.1)	0.030
Hyperlipidemia	114 (54.5)	75 (36.1)	< 0.001
Pulmonary hypertension	1 (0.4)	5 (2.6)	0.215
Pulmonary infection	26 (12.4)	69 (33.2)	< 0.001
Previous thrombosis	8 (3.8)	10 (4.8)	0.622
Previous PCI	65 (31.1)	52 (25.0)	0.166
Previous PTCA	14 (6.7)	10 (4.8)	0.407
Previous cardiac bypass	4 (1.9)	6 (2.9)	0.743
Previous cardiac surgery	0 (0.0)	6 (2.9)	0.013
Smoking	112 (53.6)	104 (50.0)	0.463
Drinking	21 (10.0)	15 (7.2)	0.302
Medication			
CCB	42 (20.1)	40 (19.2)	0.824
Antiplatelet drug	169 (80.9)	159 (76.4)	0.271
Dual antiplatelet drug	160 (76.6)	153 (73.6)	0.479
β-blockers	118 (56.5)	110 (52.9)	0.463
Statins	166 (79.4)	155 (74.5)	0.234
ACEIs/ARBs/ARNI	77 (36.8)	70 (33.7)	0.496
Oral antidiabetic drugs	185 (88.5)	192 (92.3)	0.189
Insulin treatment	57 (27.3)	58 (27.9)	0.889
Laboratory examination			
ALB, g/L	40.7 (38.9, 42.9)	35.7 (33.1, 38.3)	< 0.001
PNI	51.2 (49.2, 53.8)	42.8 (39.8, 45.2)	< 0.001
ALC, × 10^9^/L	2.1 (1.7, 2.5)	1.3 (1.0, 1.6)	< 0.001
CRP, mg/L	1.5 (1.2, 2.3)	2.2 (1.4, 3.0)	< 0.001
FBG, mmol/L	7.3 (6.2, 10.2)	7.7 (6.1, 10.8)	0.764
HbA1c, %	7.0 (6.3, 7.9)	7.3 (6.4, 8.1)	0.061
Cr, μmol/L	84.0 (68.0, 103.0)	95.0 (74.0, 149.0)	0.003
LVEF, %	63.0 (54.1, 67.4)	54.8 (43.8, 64.0)	< 0.001
BNP, pg/mL	267.0 (139.0, 367.5)	302.5 (143.0, 370.8)	0.426
UA, mmol/dL	369.0 (303.5, 445.5)	371.5 (305.0, 472.3)	0.616
TG, mmol/L	1.8 (1.3, 2.5)	1.4 (1.0, 2.0)	< 0.001
Type and number of infarcted vessels			
RCA	184 (88.0)	182 (87.5)	0.867
LMCA	41 (19.6)	54 (26.0)	0.122
LAD	197 (94.3)	197 (94.7)	0.839
LCX	169 (80.9)	169 (81.3)	0.919
Number of infarcted vessels > 2	149 (71.3)	155 (74.5)	0.458
Intervention programs (I: Drug coated stent + balloon dilation; II: Drug balloon surgery)			
Drug coat stent + balloon dilation	165 (78.9)	144 (69.2)	0.024
Number of stents placed > 1	75 (35.9)	67 (32.2)	0.429
Time intervals for imaging > 7 d	196 (93.8)	167 (80.3)	< 0.001

Continuous variates are shown as mean ± SD or median (IQR), and categorical data are expressed as proportions (n%)

*ACEIs* angiotensin-converting enzyme inhibitor, *ALB* serum albumin, *ALC* absolute lymphocyte count, *ARBs* angiotensin receptor blockers, *ARNI* angiotensin receptor neprilysin inhibitor, *BMI* body mass index, *BNP* B-type brain natriuretic peptide, *CCB* calcium channel blocker, *Cr* creatinine, *CRP* C-reactive protein, *FBG* fasting blood glucose, *HbA1c* glycosylated hemoglobin, type A1c, *LAD* left anterior descending, *LCX* left circumflex, *LMCA* left main coronary artery, *LVEF* left ventricular ejection fraction, *MACE* major adverse cardiovascular events, *PCI* percutaneous coronary intervention, *PNI* prognostic nutritional index, *PTCA* percutaneous transluminal coronary angioplasty, *RCA* right coronary artery, *TG* triglyceride, *UA* uric acid

### Effect of preoperative PNI classification on clinical outcomes

**Fig. 2:**
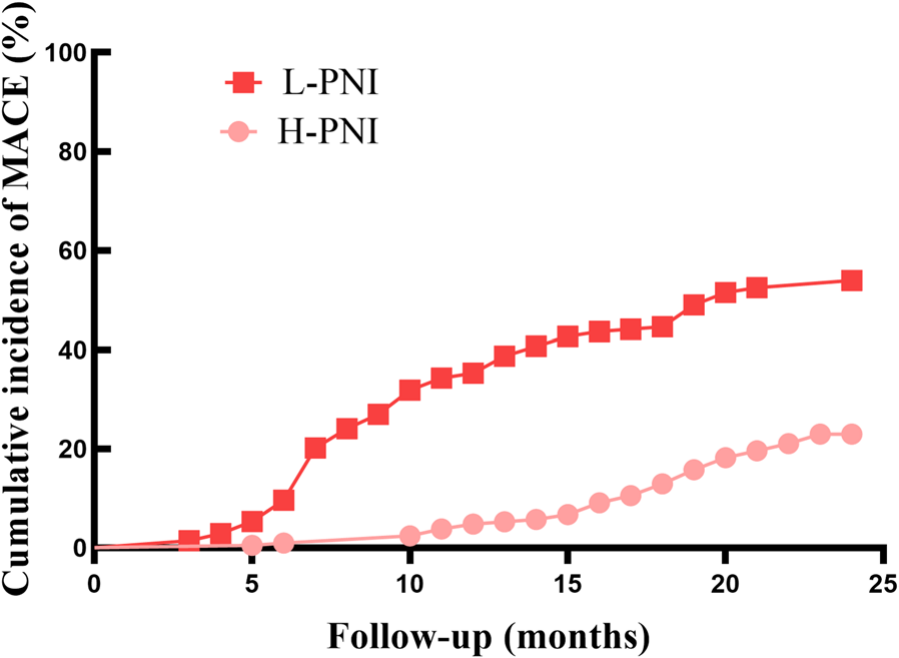
Kaplan–Meier survival estimate for MACE outcomes

**Fig. 3:**
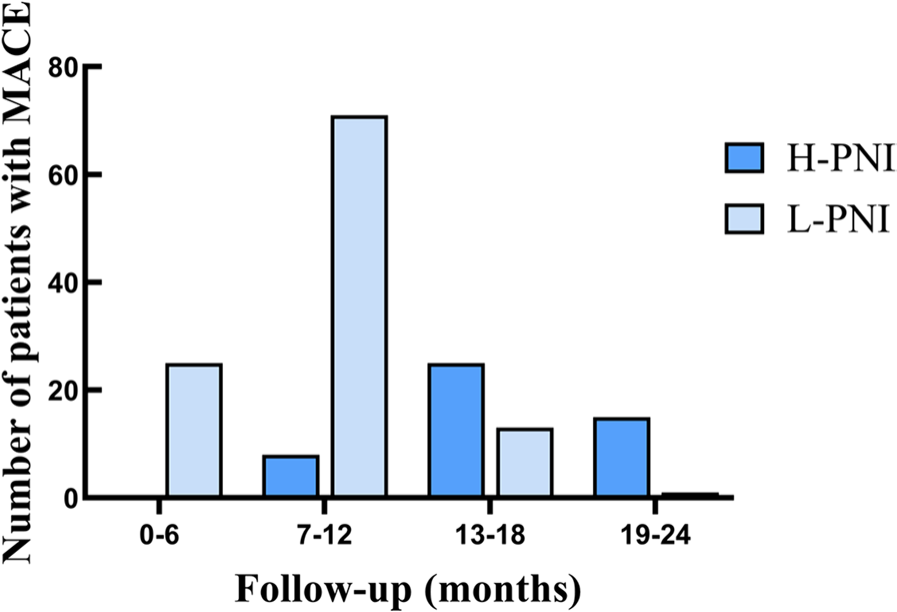
Data on the number of patients experiencing MACE over different follow-up periods (only the number of patients experiencing an adverse event for the first time)

Kaplan–Meier survival curves revealed a statistically significant difference in total MACE between the two groups (log-rank *p* < 0.001; Fig. 2). Temporally, there was a tendency for patients in the L-PNI group to develop MACE earlier than patients in the H-PNI group (Fig. 3). According to the results of Cox univariate analysis of MACE in patients in Table 2, the risk of MACE in the L-PNI group was 2.64 times higher than that in the H-PNI (95% CI 1.82–3.83, *p* < 0.001).

**Table 2:** Cox proportional hazards regression analysis for the rate of MACE in patients in the postoperative period

Variable	Univariate		Multivariate	
	HR (95% CI)	*p*	HR (95% CI)	*p*
Sex, male	1.17 (0.79, 1.73)	0.445		
Age, years	1.05 (1.03, 1.07)	< 0.001	1.04 (1.02, 1.06)	< 0.001
BMI, kg/m^2^	0.96 (0.92, 1.00)	0.062		
LVEF, %	0.96 (0.95, 0.97)	< 0.001	0.97 (0.95, 0.98)	< 0.001
Dual antiplatelet drug	1.38 (0.93, 2.04)	0.061		
Hypertension	2.73 (1.81, 4.13)	< 0.001	1.52 (1.01, 2.28)	0.044
Hyperlipidemia	1.71 (1.25, 2.34)	0.001	1.98 (1.42, 2.76)	< 0.001
CRP, mg/L	1.01 (1.01, 1.02)	< 0.001	1.01 (1.00, 1.01)	0.002
L-PNI (H-PNI as reference)	3.23 (2.30, 5.53)	< 0.001	2.64 (1.82, 3.83)	< 0.001
HbA1c, %	1.11 (0.98, 1.26)	0.108		
Cr, μmol/L	1.008 (1.006, 1.009)	< 0.001	1.005 (1.003, 1.007)	< 0.001
Pulmonary infection	2.10 (1.50, 2.94)	< 0.001	1.23 (0.85, 1.78)	0.274
Previous PCI	1.36 (0.98, 1.88)	0.067		
Smoking	1.70 (1.24, 2.35)	0.001	2.05 (1.46, 2.88)	< 0.001
Drinking	1.15 (0.68, 1.96)	0.607		
TG, mmol/L	0.95 (0.87, 1.04)	0.271		
Time intervals for imaging > 7 d	0.69 (0.45,1.05)	0.083		
Number of infarcted vessels > 2	1.15 (0.81, 1.65)	0.446		

*BMI* body mass index, *Cr* creatinine, *CRP* C-reactive protein, *HbA1c* glycosylated hemoglobin, type A1c, *LVEF* left ventricular ejection fraction, *PCI* percutaneous coronary intervention, *PNI* prognostic nutritional index, *TG* triglyceride

The analysis revealed that patients in the L-PNI group had a greater risk of MACE such as non-fatal stroke, heart failure, unstable angina, and atrial fibrillation as opposed to those in the H-PNI group. After adjusting, the risk of hospitalization for unstable angina in the L-PNI group was 2.55 times higher than that of patients in the H-PNI group (95% CI 1.570–4.140, *p* < 0.001). The L-PNI group was not associated with a risk of cardiovascular deaths before or after correcting. (Table 3).

**Table 3:** Associations of PNI level with clinical outcomes

Outcomes	Events/total	Crude HR (95% CI)	*p*	Adjusted HR (95% CI)	*p*
Cardiovascular death					
H-PNI	5/209	Reference	–	Reference	–
L-PNI	10/208	2.57 (0.88, 7.54)	0.086	0.82 (0.26, 2.63)	0.743
Non-fatal stroke					
H-PNI	19/209	Reference	–	Reference	–
L-PNI	28/208	2.06 (1.15, 3.70)	0.015	1.44 (0.76, 2.70)	0.261
Hospitalization for heart failure					
H-PNI	7/209	Reference	–	Reference	–
L-PNI	13/208	2.68 (1.07, 6.74)	0.036	2.33 (0.87, 6.26)	0.094
Hospitalization for unstable angina					
H-PNI	30/209	Reference	–	Reference	–
L-PNI	58/208	2.74 (1.76, 4.26)	< 0.001	2.55 (1.57, 4.14)	< 0.001
Atrial fibrillation					
H-PNI	15/209	Reference	–	Reference	–
L-PNI	25/208	2.34 (1.23, 4.45)	0.010	1.69 (0.84, 3.43)	0.144

*CI* confidence interval, *HR* hazard ratio, *PNI* prognostic nutritional index

## Discussion

This study represents the initial assessment of the impact of PNI levels on the prognosis of diabetic individuals with MI within two years following angiographic intervention. Patients in the L-PNI group experienced an earlier onset of MACE and had a higher overall risk of adverse events compared to those in the H-PNI group. We further correlated the categories of all MACE that had occurred in patients during the follow-up period sequentially with PNI. After adjusting for other factors, the results showed that lower PNI level was still related to an increased risk of adverse events in the category of unstable angina leading to hospital admission in the patients after the intervention. Our findings suggest that low PNI is essential for poor prognosis in the entire DM combined with the MI population.

Diabetic patients with MI comprised the sample population, representing a complex condition. First of all, DM raises the risk of malnutrition by increasing protein catabolism and reducing protein synthesis, which results in an imbalance in nitrogenous matter [6]. In one prior research, it was revealed that the prognostic impact of malnutrition was enhanced when DM was present. This is possible because albumin has a function in inhibiting autophagy. However, DM patients have elevated levels of autophagy in cardiac tissues, thereby amplifying the impacts of hypoalbuminemia [19]. Secondly, DM is characterized by two critical metabolic abnormalities: hyperglycemia and insulin resistance. Hyperglycemia leads to chronic inflammation, smooth muscle proliferation, and increased permeability of endothelial cells [5, 27, 28]. Additionally, current research has shown a link between insulin resistance and CVD in DM patients [29]. The hyperinsulinemia it causes promotes lipoprotein transport and causes fatty deposits in the arterial wall, thus leading to inflammation [30]. It has been indicated that insulin resistance associated with chronic inflammation can also induce accelerated vascular aging by enhancing telomere shortening (a marker of cellular senescence) [31]. In addition to this, the extent to which DM affects patients is related to their age [13]. Based on the above description of the disease characteristics of DM, suggests the need to strengthen the monitoring of nutritional and inflammation-immunity-related aspects in diabetic patients with CVD.

PNI, as a nutritional indicator, also can reflect the immune status of the body. In this study, patients in the L-PNI group had lower albumin levels and lymphocyte counts and were at higher risk of developing MACE, which is similar to the results of existing studies [32]. However, a notable distinction from prior research lies in the findings of the current study: patients assigned to the L-PNI group exhibited a greater likelihood of experiencing unstable angina resulting in hospital admission, even after controlling for potential confounding variables, in comparison to patients in the H-PNI group, about the risk of developing other MACE. Albumin has been demonstrated to be an effective predictor of adverse outcomes in patients with acute MI [15]. Even one year after discharge, the risk of all-cause mortality in patients with low albumin levels was nearly double that of the general population, according to the data of another Japanese study [33]. This also suggests that both short-term and long-term monitoring of albumin levels is clinically significant in patients with acute MI. Hypoalbuminemia is related to an increased risk of CVD for several reasons: firstly, albumin has anti-inflammatory and antitumor activity and antithrombotic capacity [15]; secondly, hypoalbuminemia exacerbates some CVD, and there is evidence that decreased albumin levels promote pulmonary edema and fluid retention, which ultimately result in heart failure [34]; thirdly, there is a relation between reduced albumin and the inflammatory response, as monocyte products of the inflammatory process, such as interleukin-1, promote reduced albumin synthesis [35]. Meanwhile, several studies have indicated a correlation between albumin and various inflammatory phase markers [36].

There is evidence of a U-shaped association between lymphocyte count and the risk of developing various arrhythmias [37]. Shah et al. [38] claimed that low lymphocyte counts are associated with increased short-term incidence of heart failure and coronary mortality in the general population. Our findings corroborate previous research. The reduction in lymphocytes suggests a compromised immune defense mechanism and highlights the extensive regulatory function of lymphocytes in the inflammatory process throughout all phases of atherosclerosis. Lymphocyte deficiency causes the emergence of an uncontrolled immune response and a multidirectional pro-inflammatory imbalance, ultimately leading to plaque formation [39]. It has been observed that lymphocytes play a crucial role in the healing process following MI. For instance, CD4^+^ T cells are activated in response to MI, contributing to a protective mechanism that helps avoid left chamber extension and rupture. The CD8 + AT2R + T-cell subset assists in maintaining cardiomyocyte viability while diminishing infarct size [14]. This mechanism also partly explains our findings. Likewise, in the study by Julio et al. [40], the rate of MI recurrence was higher in patients with low lymphocytes at a median follow-up time of 3 years.

### Innovations and limitations

Beyond a brief reference to dietary nutrition as a modifiable risk factor for MI recovery, the present guidelines have not fully stressed the relevance of recognizing and intervening in nutrition, as well as devising particular nutritional therapy for diabetic patients with MI [41]. Our findings support the need for increased awareness and identification of malnutrition in the care of diabetic patients with MI rather than considering BMI alone. However, the major limitation of this study is that the relatively small size of our study limits our ability to more precisely determine the prognostic impact of PNI on diabetic patients with MI after interventional procedures. The results we obtained should be confirmed by a major randomized clinical trial. Beyond that, PNI levels may be influenced by hormonal changes, such as serum catecholamines and cortisol. The postoperative follow-up duration was short, which could have led to changes in the nutritional status of patients during the 2-year monitoring period. Therefore, a more rigorous follow-up program is needed for this study.

## Conclusion

This prospective observational study demonstrates that low PNI is associated with MACE after coronary angiography intervention in diabetic patients with MI. The findings suggest that healthcare practitioners should focus on nutritional and immunological assessments of these patients. They should also actively investigate other CVD-related predictors to improve disease prognosis and clinical decision-making.

### Supplementary Information

Supplementary Material 1.

## Data Availability

No datasets were generated or analysed during the current study.

## Abbreviations

ACS: Acute coronary syndromes
ALB: Albumin
ALC: Absolute lymphocyte count
BMI: Body mass index
BNP: B-type brain natriuretic peptide
CHD: Coronary heart disease
CI: Confidence interval
CONUT: Controlling nutritional status score
Cr: Creatinine
CRP: C-reactive protein
CVD: Cardiovascular disease
DM: Diabetes mellitus
FBG: Fasting blood glucose
GNRI: Geriatric nutritional risk index
HbA1c: Glycosylated hemoglobin, type A1c
HR: Hazard ratio
LVEF: Left ventricular ejection fraction
MACE: Major adverse cardiovascular events
MI: Myocardial infarction
MNA: Mini-nutritional assessment
OGTT: Oral glucose tolerance test
PNI: Prognostic nutritional index
PTCA: Percutaneous transluminal coronary angioplasty
TG: Triglyceride
UA: Uric acid
